# Species composition and insecticide resistance status of *Anopheles gambiae* (*s.l.*) (Culicidae) in Kome, southern Chad and the implications for malaria control

**DOI:** 10.1186/s13071-016-1758-0

**Published:** 2016-08-23

**Authors:** Samuel Dadzie, Maxwell A. Appawu, Clement Kerah-Hinzoumbe, Martin C. Akogbeto, Michele Adimazoya, Demba Kodindo Israel, Amen Nakebang Fadel, Jacob Williams

**Affiliations:** 1 Noguchi Memorial Institute of Medical Research, P. O. Box LG 581, Legon, Ghana; 2 Programme National de Lutte contre le Paludisme, N’Djamena, Chad; 3 National University of Benin, Abomey, Calavi, Benin; 4 Research Triangle Institute, Washington, DC 20005-3967 USA

**Keywords:** Vector susceptibility, Insecticide resistance, Acetylcholinesterase-1, Knockdown resistance, Biochemical resistance

## Abstract

**Background:**

The development and spread of insecticide resistance among malaria vectors, is a threat to the continued effectiveness of interventions to control and eliminate the disease. The status of insecticide resistance among malaria vector populations at two sites in Kome, southern Chad, was evaluated to inform decisions on vector control.

**Methods:**

Mosquito larvae were collected from temporary rain-filled and semi-permanent breeding places at two sites and reared in a laboratory. Emerging *Anopheles gambiae* (*senso lato*) (*s.l*.) adults were morphologically identified, sorted and evaluated for susceptibility to WHOPES recommended insecticides. Standardized biomolecular and biochemical methods were used to determine sibling species and molecular forms: knockdown resistant alleles (*kdr-w*) for pyrethroids and DDT; acetylcholinesterase-1 resistant alleles for organophosphate and carbamates; biochemical resistance through measurement of the levels of non-specific esterase (α and β), oxidase and glutathione-s-transferases activities.

**Results:**

*Anopheles gambiae* (*s.l.*) was the main vector group in the two study sites and comprised of *Anopheles gambiae* (*senso stricto*) (*s.s.*) and *An. arabiensis*, respectively, at 71 and 29 % in Site A, and 60 and 40 % at Site B. *Anopheles gambiae* (*s.s.*) was composed of M (*Anopheles coluzzii*) and S [nominotypical *An. gambiae* (*s.s.*)] molecular forms. *Anopheles coluzzii* accounted for over 98 % of the sub-group. There was extensive phenotypic resistance to pyrethroids, DDT and carbamates, but full susceptibility to organophosphates. Population-wide frequency of knockdown resistant allele in *An. gambiae* (*s.l.*) was 43 homozygous (RR), 19 heterozygous (RS) and 38 % homozygous susceptible (SS). When segregated by species and molecular forms, *An. coluzzii* had the highest *kdr-w* frequency of 37.4 homozygous resistant alleles, and 17.5 % heterozygous, with 8.3 % homozygote susceptible alleles. *An. gambiae* (*s.s.*) had 1 % homozygous resistant allele. Levels of esterase, oxidase and glutathione-s-transferases were not significantly different compared to fully susceptible laboratory raised *An. gambiae* (*s.s.*) Kisumu reference, although few individuals showed significant elevation of esterases (> 0.04 μg/protein), indicating a likely start of biochemical enzyme resistance.

**Conclusions:**

There is an urgent need for action to stop and reverse significant insecticide resistance in the area. A comprehensive entomological surveillance and monitoring program is needed to understand the full extent of resistance to enable realistic insecticide resistance management strategy, and also to track future changes in the vector populations.

## Background

There have been dramatic reductions in malaria cases in many places around the world, over the last decade [1]. The development of insecticide resistance by mosquito vectors of malaria, however threatens this achievement, as it could undermine the efficacy of the mostly insecticide-based vector control interventions [2], including the current main interventions of long-lasting insecticidal nets (LLINs) and indoor residual spraying (IRS). The World Health Organization (WHO) currently recommends six pyrethroids for impregnating nets and 12 insecticides, belonging to four classes of insecticides (pyrethroids, organophosphates, carbamates and organochlorines), for IRS. The insecticides work similarly, by targeting specific sites in the nervous system on insects. Resistance arises when mutation at a target site prevents the insecticide from binding and thereby blocks the action of the insecticide. Knockdown resistance (*kdr*) results from a mutation in the sodium ion channel target site of DDT and pyrethroids, while target site resistance for carbamate and organophosphates is caused by mutation of the G119S Acetylcholinesterase gene (Ace-1^R^) [3, 4]. Biochemical or metabolic resistance is conferred by increased production of specific enzymes that metabolize or sequester the insecticide before it exerts toxic effects. Three main enzyme systems are involved in metabolic resistance: esterases, mono-oxygenases and glutathione S-transferases. Different enzymes affect different classes of insecticides.

The West Africa region is noted for elevated insecticide resistance in local mosquito vectors. Target site and metabolic resistance have been demonstrated in parts of Chad and in almost all the bordering countries [5–11]. Chad has adopted the current WHO policy of universal LLIN coverage of all populations at risk of malaria, and has been implementing large scale use of LLINs. As at 2013, over 50 % of the population reported coverage [1]. Very limited application of IRS occur in small locations in the southern part of the country. There are significant gaps in the knowledge on the malaria vectors in the country, and the levels and distribution of insecticide resistance in the vector populations have not been adequately characterized [7]. Although the epidemiological impact of pyrethroid resistance on the efficacy of LLINs and pyrethroid-based IRS, is unclear, limited studies elsewhere in West Africa point to potential reduction in efficacy [12]. Pursuant to the goals of the WHO global action plan for Insecticide resistance management [2], it is important for Chad to redouble efforts to generate adequate information on the status of insecticide resistance, to enable the development of realistic strategies to stem and reverse the development of insecticide resistance in the country. This study contributes to that effort.

## Methods

### Larval collection sites

**Fig. 1:**
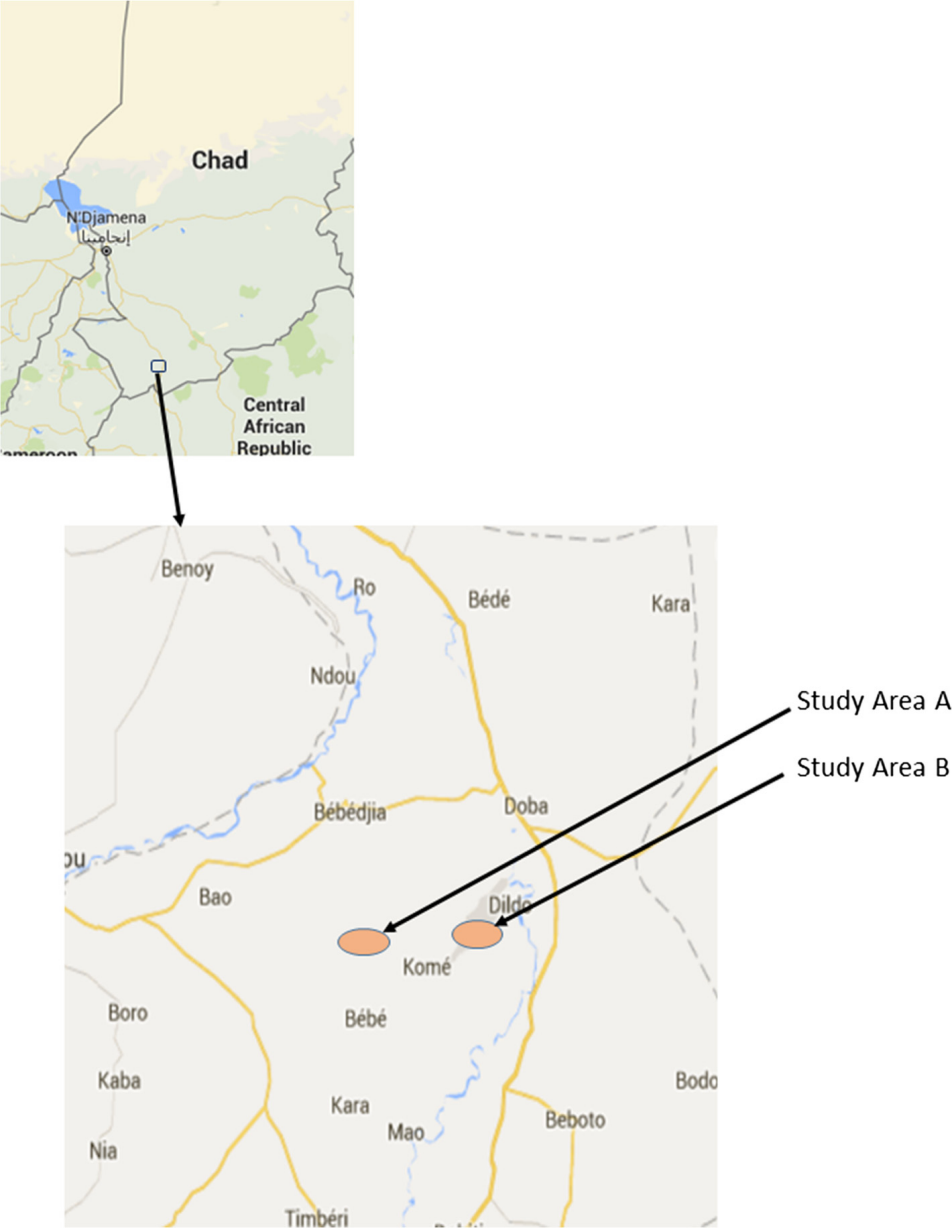
Location of the study sites in Kome, southern Chad

The study evaluated the resistance status of *An. gambiae* (*s.l.*) vector populations in two study sites in Kome: Site A (8°36′0″N, 16°26′0″E) and Site B (8°21′0″N, 15°78′0″E) in the Logone Oriental region of southern Chad (Fig. 1). The two sites are situated in an area of intensive agricultural production with cotton, rice, vegetables and oil seeds as main crops and have a mean annual rainfall of 1,200 mm and are about 16 km apart.

Each study site covered about square miles, with the two sites separated by about 8 miles. Temporary rain pools and semi-permanent breeding sites in the two sites, were sampled for mosquito larvae. Sampling took place from 15th October 2013 to 7th November 2013, during the last part of the rainy season. The larvae collected from the two sites were reared separately to adults in the laboratory at temperature of 28 ± 2 °C and relative humidity of 72 ± 5 %. Emerging adult female *An. gambiae* (*s.l.*) were morphologically identified using the taxonomic keys of Gillies & Coetzee [13], sorted out, and fed on 10 % sugar solution. These vectors were subsequently used for the evaluations described in following sections.

### Determining phenotypic resistance

The WHO tube method for insecticide susceptibility [3] was used for determining the phenotypic resistance. Non-blood fed female *An. gambiae* (*s.l.*), 2 to 5 days post-emergence, were exposed to WHO diagnostic doses of nine insecticides currently recommended for malaria vector control (deltamethrin 0.05, DDT 4.0, fenithrothion 1.0, lamdacylothrin 0.05, malathion 5, pirimiphos methyl 0.9, propoxur 0.1, bendiocarb 0.1 and permethrin 0.75 %). For each insecticide, a total of 100 *An. gambiae* (*s.l.*) were tested, comprising four replicates of 20 mosquitoes per test and a control setup of 20 mosquitoes. Twenty four-hour mortality was scored and susceptibility levels determined using WHO criteria [3].

### Vector species identification

Samples of the dead and surviving morphologically identified *An. gambiae* (*s.l.*) from the susceptibility evaluation, were identified into sibling species *An. gambiae* Giles (*senso stricto*) and *An. arabiensis*, using ribosomal DNA-polymerase chain reaction (PCR) [14]. A PCR-RFLP (restriction fragment length polymorphism) procedure described by Fanello et al. [15], was then used to separate *An. gambiae* (*s.s.*), into the M (*An. coluzzii*) and *An. gambiae* (*s.s.*) molecular forms.

### Determining target site resistance

The Hot Oligonucleotide Ligation Assay (HOLA) technique described by Lynd et al. [16], and real time PCR described by Bass et al. [17] was used to detect the presence of L1014F West Africa knockdown resistance gene *(kdr-w*) in samples of dead and surviving mosquitoes from the insecticide susceptibility tests. The presence of Ace-1^R^ alleles was identified using the protocol described by Weill et al. [18].

### Biochemical and metabolic enzyme activity

A fresh sample of non-blood fed 1–3 day-old first generation females from wild-caught *An. gambiae* (*s.l.*), were selected and killed by storing in a freezer at -20 °C in the field station, then ice-packed and transported to the laboratory and stored at -80 °C for further analysis. Kisumu strain *An. gambiae* (*s.s.*) was used as a reference for the assays. The levels of non-specific esterase (α and β), glutathione-s-transferase (GST) and mixed-function oxidase activities in the samples were quantified. *Anopheles gambiae* (*s.s.*) Kisumu susceptible strains were used as cut-off point to estimate activity levels (mean + 3 standard deviations, SD). Enzyme assays were performed using the method designed by Brodgon et al. [19]. The concentration of GSTs and acetylcholinesterase produced were calculated following methods described by Hemingway et al. [20]. An extinction coefficient of 4.39 m/M and 13.6 m/M (corrected for a path length of 0.6 cm) was used to convert absorbance to moles of product for GST and acetylcholinesterase, respectively.

## Results and discussion

### Status of phenotypic resistance

Results of the insecticide susceptibility assay for the nine insecticides tested, are presented in Table 1. The results indicate that *An. gambiae* (*s.l.*) populations at both sites are highly resistant to pyrethroids, especially to permethrin. The knockdown rates (Kd 60 min) were generally low in site A and B and ranged between 33.3–76.6 % and 24.5–76.7 %, respectively, for the pyrethroids (Table 1). Resistance was also detected for DDT with a 24 h mortality of 20 in site A and 40 % in site B. The same trend was observed for carbamates. However, all of the mosquitoes from both areas were susceptible to the organophosphate insecticides (Fenithrothion, Malathion and Pirimiphos methyl). The finding is generally consistent with the elevated and widespread resistance to pyrethroids and DDT among malaria vectors in the West Africa sub-region, which have been attributed to the historical intensive use of insecticides on cotton farms and in agriculture. The results obtained in this study add to the growing body of evidence of widespread pyrethroid resistance in Chad [6, 7]. Where insecticide susceptibility test results indicate the presence of phenotypic resistance, biomolecular tests are recommended to evaluate the underlying genetic and biological mechanisms responsible for the observed resistance [3] to inform the development of an insecticide resistance management strategy for the area.

**Table 1:** Insecticide susceptibility of *An. gambiae* (*s.l.*) from two study sites in Kome, southern Chad exposed to WHO diagnostic doses for nine insecticides (knockdown at 60 min and percent mortality at 24 h)

Insecticide	No. tested	Site A		Site B	
		% knockdown at 60 min (95 % CI)	% 24 h mortality (95 % CI)	% knockdown at 60 min (95 % CI)	% 24 h mortality (95 % CI)
Permethrin, 0.75 %	100	36.7 (35.9–37.4)	31.7 (29.0–34.3)	37.6 (36.4–38.9)	26.7 (25.9–27.4)
Lamdacyhalothrin, 0.05 %	100	33.3 (30.7–35.9)	48.3 (45.5–51.1)	25.4 (22.8–27.2)	32.2 (27.6–35.7)
Deltamethrin, 0.05 %	100	76.6 (72.7–80.5)	73.3 (69.4–77.2)	76.7 (75.9–77.4)	56.7 (55.9–57.4)
DDT, 4.0 %	100	33.3 (31.4–35.2)	20.0 (18.7–21.3)	41.7 (39.8–43.6)	40.0 (38.7–41.3)
Fenithrothion, 1.0 %	100	–	100 (100–100)	–	100 (100–100)
Malathion, 5 %	100	–	100 (100–100)	–	100 (100–100)
Pirimiphos methyl, 0.9 %	100	–	100 (100–100)	–	98.3 (97.6–99.0)
Propoxur, 0.1 %	100	–	41.7 (34.6–48.7)	–	–
Bendiocarb, 0.1 %	100	–	21.7 (19.8–23.6)	–	20.3 (19.3–21.6)

### Composition of the local population of *An. gambiae* (*s.l.*)

**Fig. 2:**
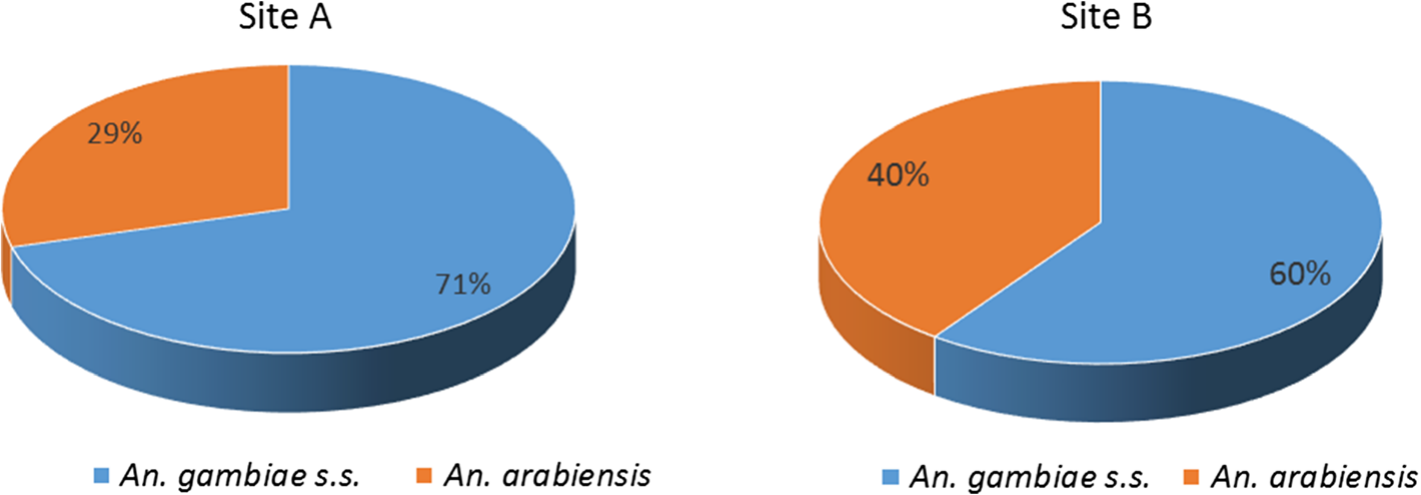
Species composition of *Anopheles gambiae* complex at two study sites in Kome, southern Chad

PCR analysis of 206 *An. gambiae* (*s.l.*) mosquitoes that were used in the insecticide susceptibility tests (107 from site A and 99 from site B), showed a dominance of *An. gambiae* Giles (*s.s.*), compared to *An. arabiensis*: 71 *vs* 29 at site A and 60 *vs* 40 % at site B (Fig. 2). The results represent a snap-shot of the population structure at the end of the rainy season. These percentages may change during course of the year, as the dramatic ecological changes from dry through rainy season impact the local vector population structure. However, the finding is similar to that previously reported by Kerah-Hinzoumbe et al. [21], which listed *An. gambiae* as the main malaria vector in south-west Chad, accounting for about 84.5 % of the annual entomological inoculation rate of 311 in that region of the country.

### Molecular forms of *Anopheles gambiae* (*s.s.*)

**Fig. 3:**
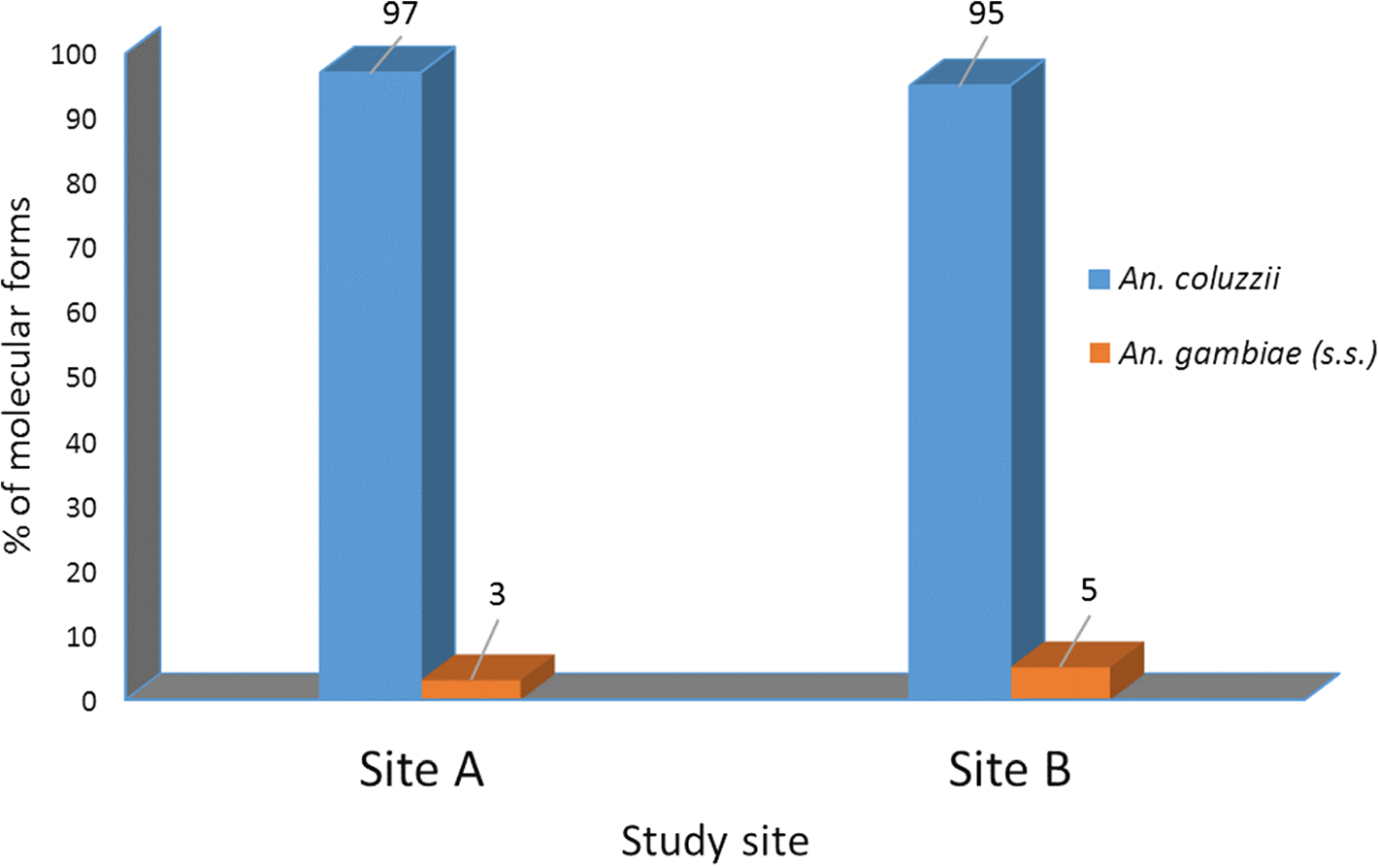
Distribution of the *An. coluzzii* (M) and *An. gambiae* (*s.s.*) molecular forms at two study sites in Kome, southern Chad

Molecular examination of 135 *An. gambiae* (*s.s.*) (76 from site A and 59 from site B) showed that the M-form (*An. coluzzii*) accounted for over 95 % of the *An. gambiae* (*s.s.*) in the two areas. The S-form, which keeps the nominotypical name *An. gambiae* (*s.s.*), occured in much smaller numbers (Fig. 3). No M/S hybrid was identified. The *An. gambiae* (*s.s.*) is widely distributed across sub-Saharan Africa, while the M-form is found only in West and Central Africa [22]. The *An. coluzzii* is thought to have a greater ability to exploit breeding sites across seasons and is more closely associated with human activities, such as rice cultivation and irrigation which tend to create semi-permanent and permanent breeding places [23]. This suggests that *An. coluzzii* may have greater ability to breed throughout the year, with a potential to sustain year-round malaria transmission. Given the significant rice cultivation and related irrigation activities in both study areas, it will be worthwhile to track changes in the molecular forms of *An. gambiae* (*s.s.*) in the area, to monitor how these correlate with future changes in land use patterns and local malaria transmission.

### Detection of knockdown resistance (*kdr-w*) gene mutation

**Fig. 4:**
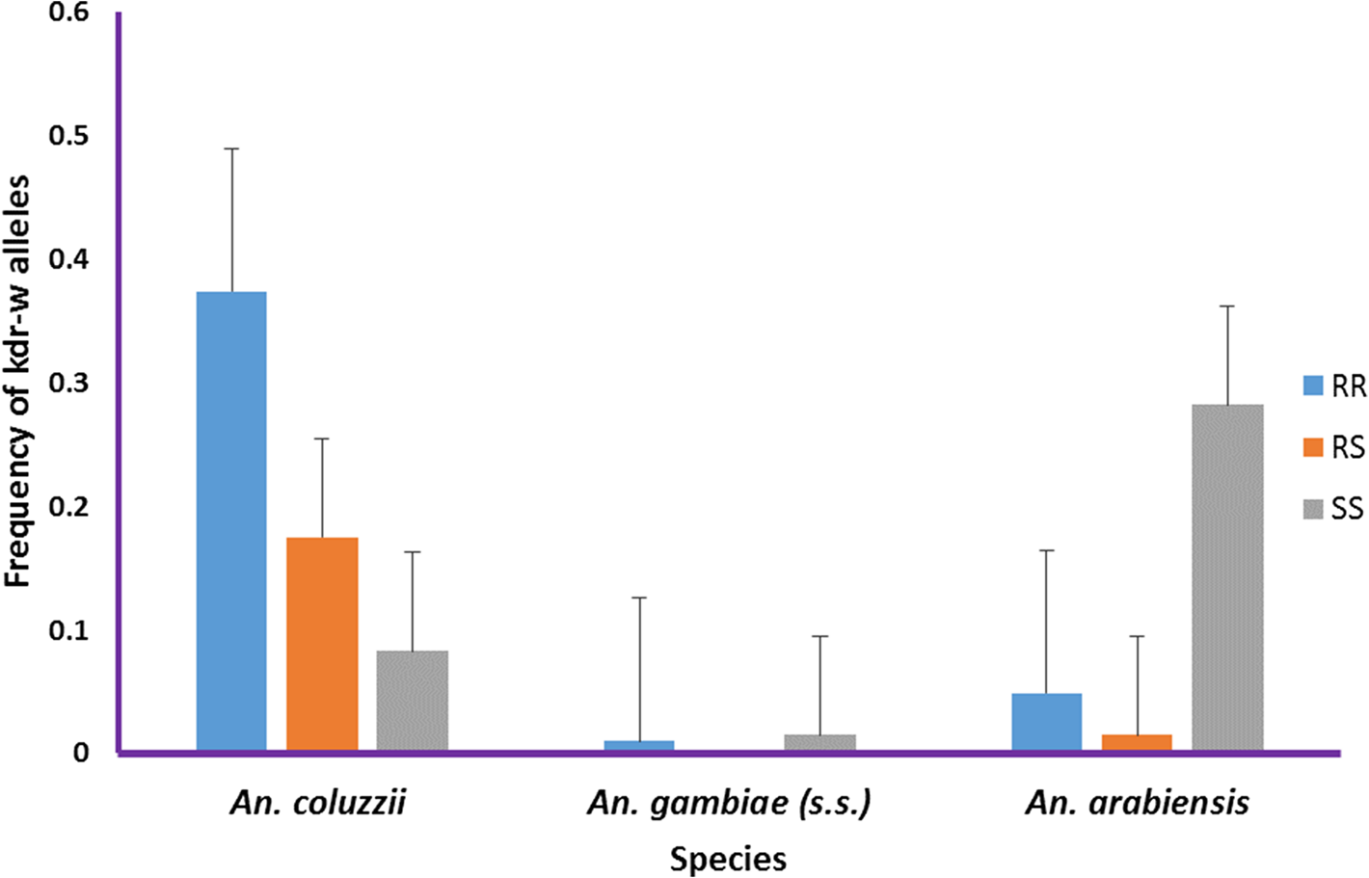
Frequency of knockdown resistant *(kdr-w*) resistant allele among *An. gambiae* complex at study sites in Kome, southern Chad. *Abbreviations*: RR, homozygote resistant allele; RS, heterozygote resistant allele; SS, homozygote susceptible allele

Analysis of a sample of 206 *An. gambiae* (*s.l.*) for *kdr-w* showed that irrespective of susceptibility status, 43 were homozygous for the resistant allele (RR), 19 as heterozygous (RS) and 38 % as homozygous susceptible alleles (SS). Segregated by species and molecular forms the allele predominantly occurred in *An. coluzzii*, with 37.4 being homozygous for site mutation, 17.5 heterozygous and about 8.3 % having no mutation (Fig. 4). Allele frequency was significantly low, 5 % (*n* = 206) homozygous resistance genotype in *An. gambiae* (*s.s.*) compared to *An. coluzzii* (Pearson Chi-square test: *χ*^*2*^ = 70.7, *df* = 1, *P* < 0.0001).

The results add to the growing evidence that *kdr* mutations may not be homogeneously distributed in *An. coluzzii* and *An. gambiae* (*s.s.*) molecular forms of *An. gambiae* (*s.s.*) [24]. We postulate that *An. coluzzii* probably face comparably higher selection pressure for *kdr*, from agriculture and related human activities, as a result of sustained presence throughout the year. The *kdr-w* resistance allele was also present in *An. arabiensis* (*n* = 206) with more homozygote susceptible (SS) alleles observed (Fig. 4).

As anticipated, there was a significantly (Pearson Chi-square test: *χ*^*2*^ = 38, *df* = 1, *P* < 0.0001) higher frequency of *kdr-w* allele among the mosquitoes that survived the susceptibility tests, while those that were killed mostly had susceptible genes. The presence of about 36 % heterozygous *kdr-w* in the individuals that survived the WHO susceptibility tests, however indicates ongoing development of resistance and thus, a need to arrest a further spread of the resistance.

There is evidence suggesting that *An. arabiensis* maintains a permanent population spread over a large area in West Africa, with a potential high level of gene flow [25]. This means that insecticide resistance genes could potentially spread rapidly within the country and sub-region. Therefore, although the current frequency of *kdr-w* in *An. arabiensis* in the Kome area is low, it is nonetheless present and could increase and spread rapidly, if effective management strategies are not put in place.

### Acetylcholinesterase target site mutation

Of all the total 137 individuals tested, the homozygous susceptible Ace-1^R^ allele was the most predominant. The same result was obtained for all categories investigated including *An. arabiensis*, both *An. coluzzii* and *An. gambiae* (*s.s.*), and status of the *kdr-w* allele (homozygous, heterozygous or resistant) and irrespective of whether or not they survived the WHO insecticide susceptibility test. Recalling that the vectors were fully susceptible to organophosphates while resistant to carbamates (negative correlation), the absence of cross resistance denotes a likelihood that the carbamate resistance was due to a mechanism other than Ace-1^R^ target site mutation and that metabolic resistance may be the main mechanism involved in the development of resistance. Further evaluation is recommended.

### Metabolic and biochemical resistance

Samples from both study sites showed a general higher activity for α esterase and oxidase, but not for GSTs, although the elevations were not significantly different from the levels of the fully susceptible *An. gambiae* (*s.s.*) Kisumu reference. In comparing site A to site B, the mean activities for α esterase and oxidase in site B were consistently higher than site A but these were not statistically significant (*P* = 0.209 and *P* = 0.408, respectively, Table 2). The activities of GST in both sites were lower than that of the Kisumu strain (0.21 ± 0.11 *vs* 0.19 ± 0.08 for α esterase; 0.12 ± 0.09 *vs* 0.11 ± 0.05 for oxidases; 0.06 ± 0.024 *vs* 0.048 ± 0.027 for GST; Table 2). While the population of mosquitoes sampled generally did not show any significant elevation of enzymes that are responsible for detoxification of insecticides, a few individuals showed significant increased activity of the enzymes, especially the esterases. Although, the mosquitoes showed high phenotypic resistance to carbamates, the frequency of the Ace-1^R^ homozygote resistance allele (RR) was low in the population. We postulate that apart from the esterases that showed increased activity, other enzymes such as CYP450 may be involved in the development of resistance. However, these other enzymes were not tested in this study due to logistical constrains.

**Table 2:** Enzyme activity levels in *An. gambiae* (*s.l.*) samples from site A and B in Kome, southern Chad

Site	Number of vectors tested	α esterase (μmol/min/mg protein)	Oxidase (μmol/min/mg protein)	GST (μmol/min/mg protein)
		Mean ± SD	Mean ± SD	Mean ± SD
Site A	76	0.19 ± 0.08 (*P* = 0.496)	0.11 ± 0.05 (*P* = 0.211)	0.048 ± 0.027
Site B	72	0.21 ± 0.11 (*P* = 0.084)	0.12 ± 0.09 (*P* = 0.097)	0.06 ± 0.024
*An. gambiae* (*s.s.*) Kisumu reference	56	0.18 ± 0.085	0.1 ± 0.041	0.08 ± 0.037

The fact a few individual mosquitoes showed elevated levels of esterases may indicate that, biochemical mechanisms may be contributing, alongside the knockdown resistant gene in the phenotypic expression of resistance. Mixed function oxidases may be acting together with *kdr* to create pyrethroid resistance. The phenomenon has been linked to intervention failure involving *An. coluzzii* elsewhere in West Africa [26, 27]. The enzyme level activity of the vectors in Kome area must be carefully monitored because of this potential threat.

## Conclusions

There is an urgent need for comprehensive entomological surveillance and monitoring program in south-western Chad, to inform the development and implementation of an effective insecticide resistance management strategy. The high level of resistance among the local malaria vectors in the area for three out of the four groups of WHOPES recommended insecticides, threatens the achievement of malaria control objectives. In particular, the significant resistance to LLIN insecticides in an area targeting universal LLIN coverage must elicit renewed efforts by national and developmental partners to address the problem. Immediate actions include active promotion of judicious use of insecticides, particularly in agriculture, to reduce the selective pressure; concerted evaluation of the utility of LLINs with insecticide-synergist combinations, which have been shown elsewhere to reduce pyrethroid resistance in *An. gambiae* [27] and is now recommended by WHO under certain conditions. The coinciding occurrence of a complete absence of Ace-1^R^ gene mutation, in the presence of settled carbamate resistance, but full susceptibility to organophosphate, adds to the growing evidence of the complexity of the interplay of biochemical, biological and gene target site mutation in the phenotypic expression of resistance.

## References

[1] WHO (2014). World malaria report 2015.

[2] WHO (2012). Global plan for insecticide resistance management in malaria vectors (GPIRM).

[3] WHO (2013). Test procedures for insecticide resistance monitoring in malaria vector mosquitoes.

[4] WHO (1981). Instructions for determining the susceptibility or resistance of mosquito larvae to insecticides.

[5] Witzig C, Parry M, Morgan JC, Irving H, Steven A, Cuamba N (2013). Genetic mapping identifies a major locus spanning P450 clusters associated with pyrethroid resistance in kdr-free *Anopheles arabiensis* from Chad. Heredity.

[6] Ranson H, Abdallah H, Badolo A, Guelbeogo WM, Kerah-Hinzoumbé C, Yangalbé-Kalnoné E (2009). Insecticide resistance in *Anopheles gambiae*: data from the first year of a multi-country study highlight the extent of the problem. Malar J.

[7] Kerah-Hinzoumbe C, Peka M, Nwane P, Donan-Gouni I, Etang J, Same-Ekobo A (2008). Insecticide resistance in *Anopheles gambiae* from south-western Chad. Central Africa Malar J.

[8] Etang J, Fondjo E, Chandre F, Morlais I, Brengues C, Nwane P (2006). First report of knockdown mutations in the malaria vector *Anopheles gambiae* from Cameroon. Am J Trop Med Hyg.

[9] Chouaïbou M, Etang J, Brevault T, Nwane P, Hinzoumbé CK, Mimpfoundi R (2008). Dynamics of insecticide resistance in the malaria vector *Anopheles gambiae* s.l. from an area of extensive cotton cultivation in Northern Cameroon. Trop Med Int Health.

[10] Kristan M, Fleischmann H, Della Torre A, Stich A, Curtis CF (2003). Pyrethroid resistance/susceptibility and differential urban/rural distribution of *Anopheles arabiensis* and *An. gambiae* (*s.s.*) malaria vectors in Nigeria and Ghana. Med Vet Entomol.

[11] Abdalla H, Matambo TS, Koekemoer LL, Mnzava AP, Hunt RH, Coetzee M (2008). Insecticide susceptibility and vector status of natural populations of *Anopheles arabiensis* from Sudan. Trans R Soc Trop Med Hyg.

[12] Agossa FR, Gnanguenon V, Anagonou R, Azondekon R, Aizoun N, Sovi A (2015). Impact of insecticide resistance on the effectiveness of pyrethroid-based malaria vectors control tools in Benin: decreased toxicity and repellent effect. PLoS One.

[13] Gillies MT, Coetzee M (1987). A supplement to the Anophelinae of Africa south of the Sahara. South African Institute for Medical Research.

[14] Scott JA, Brogdon WG, Collins FH (1993). Identification of single specimens of the *Anopheles gambiae* complex by the polymerase chain reaction. Am J Trop Med Hyg.

[15] Fanello C, Santolamazza F, Torre AD (2002). Simultaneous identification of species and molecular forms of the *Anopheles gambiae* complex by PCR-RFLP. Med Vet Entomol.

[16] Lynd A, Ranson H, McCall PJ, Randle NP, Black WC, Walker ED (2005). A simplified high-throughput method for pyrethroid knock-down resistance (kdr) detection in *Anopheles gambiae*. Malar J.

[17] Bass C, Williamson MS, Wilding CS, Donnelly MJ, Field LM (2007). Identification of the main malaria vectors in the *Anopheles gambiae* species complex using a TaqMan real-time PCR assay. Malar J.

[18] Weill M, Malcolm C, Chandre F, Mogensen K, Berthomieu A, Marquine M (2004). The unique mutation in ace-1^R^ giving high insecticide resistance is easily detectable in mosquito vectors. Insect Mol Biol.

[19] CDC (2014). Methods in *Anopheles* Research.

[20] Hemingway J, Karunaratne S (1988). Mosquito carboxylesterases: a review of the molecular biology and biochemistry of a major insecticide resistance mechanism. Med Vet Entomol.

[21] Kerah-Hinzoumbe C, Peka M, Antonio-Nkondjio C, Donan-Gouni I, Awono-Ambene P, Same-Ekobo A (2009). Malaria vectors and transmission dynamics in Goulmoun, a rural city in south-western Chad. BMC Infect Dis.

[22] Gnémé A, Guelbéogo WM, Riehl MM, Sanou A, Traoré A, Zongo S (2013). Equivalent susceptibility of *Anopheles gambiae* M and S molecular forms and *Anopheles arabiensis* to *Plasmodium falciparum* infection in Burkina Faso. Malar J.

[23] Gimonneau G, Bouyer J, Morand S, Besansky N, Diabate A, Simard F (2010). A behavioral mechanism underlying ecological divergence in the malaria mosquito *Anopheles gambiae*. Behav Ecol.

[24] Santolamazza F, Calzetta M, Etan J, Barrese E, Dia I, Caccone A (2008). Distribution of knock-down resistance mutations in *Anopheles gambiae* molecular forms in west and west-central Africa. Malar J.

[25] Simard F, Lehmann T, Lemasson J-J, Diatta M, Fontenille D (2000). Persistence of *Anopheles arabiensis* during the severe dry season conditions in Senegal: an indirect approach using microsatellite loci. Insect Mol Biol.

[26] N'Guessan R, Corbel V, Akogbeto M, Rowland M (2007). Reduced efficacy of insecticide-treated nets and indoor residual spraying for malaria control in pyrethroid resistance area. Benin Emerg Infect Dis.

[27] Corbel V, N'Guessan R, Brengues C, Chandre F, Djogbenou L, Martin T (2007). Multiple insecticide resistance mechanisms in *Anopheles gambiae* and *Culex quinquefasciatus* from Benin, West Africa. Acta Trop.

